# Over‐expression of Dyrk1A affects bleeding by modulating plasma fibronectin and fibrinogen level in mice

**DOI:** 10.1111/jcmm.17817

**Published:** 2023-07-06

**Authors:** Guillaume Postic, Jean Solarz, Cécile Loubière, Janany Kandiah, Jaysen Sawmynaden, Frederic Adam, Marie Vilaire, Thibaut Léger, Jean‐Michel Camadro, Daniella Balduino Victorino, Marie‐Claude Potier, Eric Bun, Gautier Moroy, Alexandre Kauskot, Olivier Christophe, Nathalie Janel

**Affiliations:** ^1^ Université Paris Cité, BFA, UMR 8251, CNRS, ERLU1133 Paris France; ^2^ HITh, UMR_S1176, Institut National de la Santé et de la Recherche Médicale, Université Paris‐Saclay le Kremlin‐Bicêtre France; ^3^ Université Paris Cité, BFA, UMR 8251, CNRS Paris France; ^4^ Institut Médical Jérôme Lejeune Paris France; ^5^ Université Paris Cité, IJM, UMR 7592, CNRS Paris France; ^6^ Toxicology of Contaminants Unit, Fougeres Laboratory, French Agency for Food, Environmental and Occupational Health & Safety (ANSES) Fougeres France; ^7^ ICM Paris Brain Institute, CNRS UMR7225, INSERM U1127, Sorbonne University, Hôpital de la Pitié‐Salpêtrière Paris France

**Keywords:** bleeding time, Dyrk1A, fibrinogen, fibronectin, filamin a, mice, platelet

## Abstract

Down syndrome is the most common chromosomal abnormality in humans. Patients with Down syndrome have hematologic disorders, including mild to moderate thrombocytopenia. In case of Down syndrome, thrombocytopenia is not associated with bleeding, and it remains poorly characterized regarding molecular mechanisms. We investigated the effects of overexpression of Dyrk1A, an important factor contributing to some major Down syndrome phenotypes, on platelet number and bleeding in mice. Mice overexpressing Dyrk1A have a decrease in platelet number by 20%. However, bleeding time was found to be reduced by 50%. The thrombocytopenia and the decreased bleeding time observed were not associated to an abnormal platelet receptors expression, to a defect of platelet activation by ADP, thrombin or convulxin, to the presence of activated platelets in the circulation or to an abnormal half‐life of the platelets. To propose molecular mechanisms explaining this discrepancy, we performed a network analysis of Dyrk1A interactome and demonstrated that Dyrk1A, fibronectin and fibrinogen interact indirectly through two distinct clusters of proteins. Moreover, in mice overexpressing Dyrk1A, increased plasma fibronectin and fibrinogen levels were found, linked to an increase of the hepatic fibrinogen production. Our results indicate that overexpression of Dyrk1A in mice induces decreased bleeding consistent with increased plasma fibronectin and fibrinogen levels, revealing a new role of Dyrk1A depending on its indirect interaction with these two proteins.

## 1 INTRODUCTION

Down syndrome (DS) or trisomy 21 is the most common chromosomal abnormality in humans. DS is a complex genetic condition due to an extra copy of human chromosome 21 (HSA21) resulting in many genes deregulation.^1^ DS is associated with multiple disease spectrum.^1^ Among them, all individuals with DS exhibit mild to severe intellectual disability.^2^ Patients with DS also have hematologic disorders, including thrombocytopenia, which is seen in 66% of DS babies.^3^, ^4^ Even if thrombocytopenia is mild to moderate and not associated with bleeding, it remains poorly characterized regarding molecular mechanisms.

Dual specificity Tyrosine (Y) phosphorylation‐Regulated Kinase 1A (Dyrk1A) encodes a proline‐directed serine/threonine kinase, whose gene is located on HSA21 in the DS critical region. Dyrk1A is an important factor contributing to intellectual disability, memory deficit and Alzheimer's disease (AD)‐type dementia, the main features of the DS phenotypes.^5^, ^6^ The key functions of Dyrk1A have been well documented in neuronal defects observed in DS patients.^7^ With its location within the DS critical region, Dyrk1A has been suggested to play a crucial role in brain alterations both in trisomy and monosomy 21 patients.^8^ Minimal deletions overlapping 21q22 microdeletion containing among others the *Dyrk1A* and *RUNX1* gene and covers the DS critical region have been described in partial monosomy 21 patients.^9^, ^10^ Interestingly, these patients present not only intellectual disability but also idiopathic thrombocytopenia. Shinawi et al^11^ described platelet pool storage disease caused by *RUNX1* haploinsufficiency and multiple problems of microdeletions at 21q22 ascribed to *RUNX1* and *Dyrk1A*. Surprisingly, patients with partial 21 monosomy were diagnosed with low platelet counts, although *RUNX1* is not deleted.^10^ Therefore, we hypothesized that Dyrk1A may also affect platelet counts and bleeding. More recently to explore differences in the circulating proteomes of DS individuals, Sullivan and colleagues compared a series of studies focusing on secreted proteins and those with extracellular domains.^12^ 299 proteins were differentially detected between the plasma of DS individual versus euploid controls. Among them, multiple proteins within the response to wounding/regulation of coagulation were identified in DS samples, including fibrinogen.^12^ Fibrinogen is a plasma protein involved in primary and secondary haemostasis, thus playing a crucial role in clot formation. We therefore assessed Dyrk1A interaction with proteins extracted from Epstein–Barr virus‐transformed lymphoblastoid cell lines (LCLs) of healthy individuals and unrelated individuals with DS and investigated the effects of Dyrk1A overexpression on plasma fibronectin and fibrinogen, platelet number and half‐life, platelet receptors expression, platelet activation and bleeding in mice.

## 2 METHODS

### 2.1 Experimental mice

All procedures were carried out in accordance with the ethical standards of French and European regulations (European Communities Council Directive, 86/609/EEC). Official authorization from the French Ministry of Agriculture was granted to perform research and experiments on animals (authorization number 75–369) and the experimental protocol was approved by the institutional animal care and use committee of the Paris Diderot University (CEEA40). Mice were housed in a controlled environment with unlimited access to food and water on a 12‐h light/dark cycle. The number of mice used, and suffering were minimized as much as possible. The murine bacterial artificial chromosome 189 N3 (mBACtgDyrk1A) strain was previously constructed by electroporating HM‐1 embryonic stem cells with the retrofitted BAC‐189 N3.^13^ The mBACtgDyrk1A (TgDyrk1A) and Dp (16)1Yey mice were maintained on a C57Bl/6J background and genotyped as described.^13^, ^14^ Male and female mice from the same litter, 3 months of age, were used. Number and suffering of mice being reduced as much as possible.

### 2.2 Cell lines, culture conditions and protein analysis

LCLs were derived from B lymphocytes of three healthy individuals and three unrelated individuals with DS as described.^15^ LCLs are easy to grow and are widely used to study genotype–phenotype correlation.^16^ Parents of patients from the Institut Jérôme Lejeune gave their informed consent, and the French biomedical ethics committee gave its approval for this study (Comité de Protection des Personnes dans la Recherche Biomédicale number 03025). LCLs were cultured in Opti‐MEM with GlutaMax (Invitrogen, Cergy, France) supplemented with 5% fetal bovine serum from a unique batch and 1% penicillin and streptomycin mix (10,000 U/mL). Cell lines were grown at 37°C in humidified incubators under 5% CO2. Cells were harvested by centrifugation, were washed in PBS, followed by another centrifugation and were stored at −80°C. Cell lysates were obtained from 5 × 10^6^ cell pellets treated with 300 μL of lysis buffer [Tris, 50 mM, pH 8; NaCl, 150 mM; Igepal, 1% (Sigma‐Aldrich, France); SDS, 0.1% containing protease inhibitors (1 mM Pefabloc SC, 5 μg/mL E64 and 2.5 μg/mL Leupeptin). After centrifugation for 10 min at 15,000 × g at 4°C, the cell lysate was stored at −80°C. To assess Dyrk1A interaction with proteins, co‐IP experiments were performed with 200 μg of protein incubated 2 h with 2 μg rAb anti‐Dyrk1A (Abnova) at 4°C. The immunocomplexes were incubated with protein A/G Magnetic beads (Thermo Scientific Pierce; Fisher Scientific SAS, Illkirch, France) overnight at 4°C, and beads were washed. Dyrk1A co‐IP was analysed by mass spectrometry (MS). All tryptic digests of protein extracts were analysed with a LTQ Velos Orbitrap equipped with an EASY‐Spray nanoelectrospray ion source and coupled to an Easy nano‐LC Proxeon 1000 system (all devices were obtained from Thermo Fisher Scientific, San Jose, CA) and chromatographic separation of the peptides. All the experimental conditions for LC–MS/MSMS acquisitions were as previously described.^17^ MS/MS data were processed with an in‐house Mascot search server (Matrix Science, Boston, MA; version 2.4.1). The mass tolerance was set to 7 ppm for precursor ions and 0.5 Da for fragments. The maximum number of missed trypsin cleavages was limited to two. The SwissProt database with the Homo sapiens taxonomy was used for the MS/MS identification step.

### 2.3 Network analysis of Dyrk1A interactome

The affinity purification‐mass spectrometry experiment produced a list of proteins that are expected to interact with Dyrk1A, either directly or indirectly. Therefore, to restore the connectivity in the Dyrk1A interactome, we submitted the list of candidate partners to the Proteo3Dnet web server.^18^ Briefly, the method finds protein–protein interactions (PPIs) by integrating data from three sources: the Protein Data Bank (PDB),^19^ the Eukaryotic Linear Motif (ELM) resource,^20^ and the Biological General Repository for Interaction Datasets (BioGRID) database.^21^ For a pair of candidate partners, the program searches for experimental structures in the PDB that gather both proteins. For case when no multimeric structure can be found, a distant homology search is performed: if both proteins have homologues located in the same 3D complex, they are then predicted as potential interactants (also called ‘interologs’), providing that the interaction has been conserved throughout evolution. The ELM database is exploited by searching for the presence of a short linear motif (SLiM) in one protein, and for the corresponding Pfam domain in the other. The two candidate partners are thus expected to interact in a transient manner. The detection is conditioned to the location of the SLiM within an intrinsically disordered region, as predicted by the IUPred2A software.^22^ Experimental proteomics data from BioGRID are also integrated to add nodes to the network, which may serve as (indirect) connections between the input proteins. This BioGRID analysis only keeps the most robust experimental data, by excluding those associated with the ‘Far Western’, ‘Co‐fractionation’, ‘Co‐localization’, ‘Biochemical Activity’ and ‘High Throughput’ experimental systems. The default parameters have been used for Proteo3Dnet which thus adds 15 nodes from BioGRID. In the results presented below, the proteins are designated by their UniProtID from which “_HUMAN” has been truncated.

### 2.4 Preparation of serum samples, protein extraction and ELISA essays

Mice were anaesthetised with ketamine/xylazine (100 mg/kg and 10 mg/kg, respectively) and blood samples were obtained by retro‐orbital sinus sampling with heparinized capillaries, collected into tubes containing an 1/10 volume of 3.8% sodium citrate. The plasma was isolated by centrifugation (1500 g) for 15 min at R.T. and kept at −80°c until use. Mice were sacrificed, and the liver dissected and stored at −80°C until analysis. The plasma Dyrk1A levels were assessed by a solid phase immobilized epitope immunoassay, as described.^23^ Plasma and liver fibrinogen (Abcam ELISA kit (# ab213478, Paris, France)), plasma fibronectin (Abcam ELISA kit ((# ab108849, Paris, France))), plasma C‐reactive protein (CRP) (Abcam ELISA kit ((# ab222511, Paris, France))), plasma Interleukin‐6 (IL‐6) (Abcam ELISA kit ((# ab222503, Paris, France))) were assessed using sandwich ELISA. After removal of unbound conjugates, bound enzyme activity was assessed by use of a chromogenic substrate for measurement at 450 nm by a microplate reader (Flex Station 3, Molecular Devices, Ltd., Wokhingham, UK).

### 2.5 Platelet counts and volume

Blood sampling was performed under anaesthesia (ketamine/xylazine (100 mg/kg and 10 mg/kg, respectively)) and blood counts were determined with an automatic cell counter (Scil Vet ABC Plus, Horiba Medical, France)

### 2.6 Harmine treatment

Mice were injected intraperitoneally (without anaesthesia) in the evening overnight, with 10 mg/kg of harmine hydrochloride hydrate (Fisher Scientific, Illkirch, France) dissolved in 0.9% NaCl. The next morning, mice were injected once more for 1 h. Control mice were injected with 0.9% NaCl (Vehicle).

### 2.7 Measure of tail bleeding time

Tail bleeding time was measured by 3 mm tail‐tip cut in anaesthetised mice (ketamine/xylazine (100 mg/kg and 10 mg/kg, respectively)). After cutting, the tail was immediately immersed in 50 mL of 0.9% sodium chloride at 37°C. A stopwatch was started immediately to determine the time required for the bleeding to stop. To ensure that bleeding did not start again, we monitored for at least 60 sec the tail bleeding. Tail bleeding assays were stopped at 600 sec if the bleeding did not stop

### 2.8 Isolation of mouse platelets, flow cytometry and western blotting

Mice were anaesthetised by intraperitoneal injection of xylazin (10 mg/kg) and ketamine (100 mg/kg), then whole blood was collected by cardiac puncture. Quantification of αIIbβ3 activation in conditions of unstirred platelets in whole blood was assessed by flow cytometry using the JON/A monoclonal antibody specific for the activated form of αIIbβ3. Isolated platelets were obtained as previously described.^24^ Platelet surface β‐galactose exposure was determined using fluorescein isothiocyanate (FITC)‐conjugated RCA or ECL (RCA #FL‐1081; ECL #FL‐1141; Vector Laboratories) as described.^25^ Washed platelets (3 × 10^8^/mL) were stimulated with a range of activators (convulxin, ADP, or thrombin). After incubation for 10 min without stirring, platelets were diluted at 10^8^/mL and incubated with the appropriate fluorophore‐conjugated antibodies for 20 min at room temperature (CD41/61‐FITC (#M021‐1, Emfret), GPIbβ‐FITC (#M040‐1, Emfret), GPIba‐FITC (#M040‐1Emfret), GPVI‐FITC (#M011‐1, Emfret, #JAQ1)). The samples were directly analysed with an Accuri C6 + flow cytometer (BD Biosciences). Platelets were lysed in denaturing buffer (50 mM Tris, 100 mM NaCl, 50 mM NaF, 5 mM EDTA, 40 mM β‐glycerophosphate, 100 μM phenylarsine oxide, 1% sodium dodecyl sulfate, 5 μg/mL leupeptin, 10 μg/mL aprotinin, pH 7.4). Proteins were separated by SDS‐polyacrylamide gel electrophoresis and transferred to nitrocellulose membranes. Membranes were incubated with the antibody directed against fibrinogen (1/1000; proteintech #20645‐1‐AP), or filaminA (1/1000; abcam #ab51217), or Dyrk1A (1/1000; Abnova #H00001859‐M01), or β‐actin (1/10000; R&D #MAB8929), or 14–3‐3 zeta (1/1000; Proteintech #14881‐1‐AP), followed by horseradish‐peroxidase‐labelled secondary antibodies. Immunoreactive bands were visualized with enhanced chemiluminescence detection reagents (Perbio Science) using a G:BOX Chemi XT16 Image System and quantified using Gene Tools version 4.03.05.0 (Syngene).

### 2.9 Platelet depletion

Mice were injected intraperitoneally (without anaesthesia) with the R300 antibody (Emfret, Eibelstadt, Germany) directed against GPIB (1 mg/g) in the evening and platelets counts were determined the next morning. C301 antibody (Emfret, Eibelstadt, Germany) was used as control.

### 2.10 Human platelet isolation and protein analysis

Human blood was collected on acid citrate dextrose solution and washed platelets were obtained by standard procedures within 2–3 h.^26^ All patients were recruited in France, informed about the anonymous use of their data and gave written informed consent in accordance with the Declaration of Helsinki. Platelet lysates were prepared in lysis buffer with a cocktail of proteases and phosphatases inhibitors. Protein concentrations were determined with the Bio‐Rad Protein Assay reagent (Bio‐Rad). To assess the relative amount of Dyrk1A, 200 mg of proteins were incubated 2 h at 4°C with 2 μg rAb anti‐Dyrk1A 7D10 (Abnova corporation, Tebu, France). The immunocomplexes were incubated with protein A‐Sepharose protein G‐ Sepharose overnight at 4°C and the beads were washed. Then the immunoprecipitates were resuspended and subjected to SDS electrophoresis on acrylamide gels under reducing conditions and transferred to Hybond‐C Extra membrane (GE Healthcare Europe GmbH, Saclay, France). After transfer, membranes were saturated by incubation in 5%w/v nonfat milk powder in Tris–saline buffer (1.5 mM Tris base pH 8; 5 mM NaCl; 0.1% Tween‐20) and incubated overnight at 4°C with the antibody directed against Dyrk1A (1/1000; Abnova #H00001859‐M01). Binding of the primary antibody was detected by incubation with the horseradish peroxidase‐conjugated secondary antibody using the Western Blotting Luminol Reagent (Santa Cruz Biotechnology, Tebu, France). Digitized images of the immunoblots were obtained using a LAS‐3000 imaging system (Fuji Photo Film Co., Ltd.).

### 2.11 Data analysis

Statistical analysis was done with the Student's *t*‐test using Statview software (Statview 3, Abacus Corporation). For multiple pairwise comparisons between genotypes and treatments, statistical analysis was done with two‐way anova followed by the Bonferroni/Dunnet post hoc test using Statview software. The results are expressed as mean ± SEM (standard error of the mean). Data were considered significant when *p* < 0.05.

## 3 RESULTS

### 3.1 Dyrk1A, fibronectin and fibrinogen interact indirectly

**FIGURE 1:**
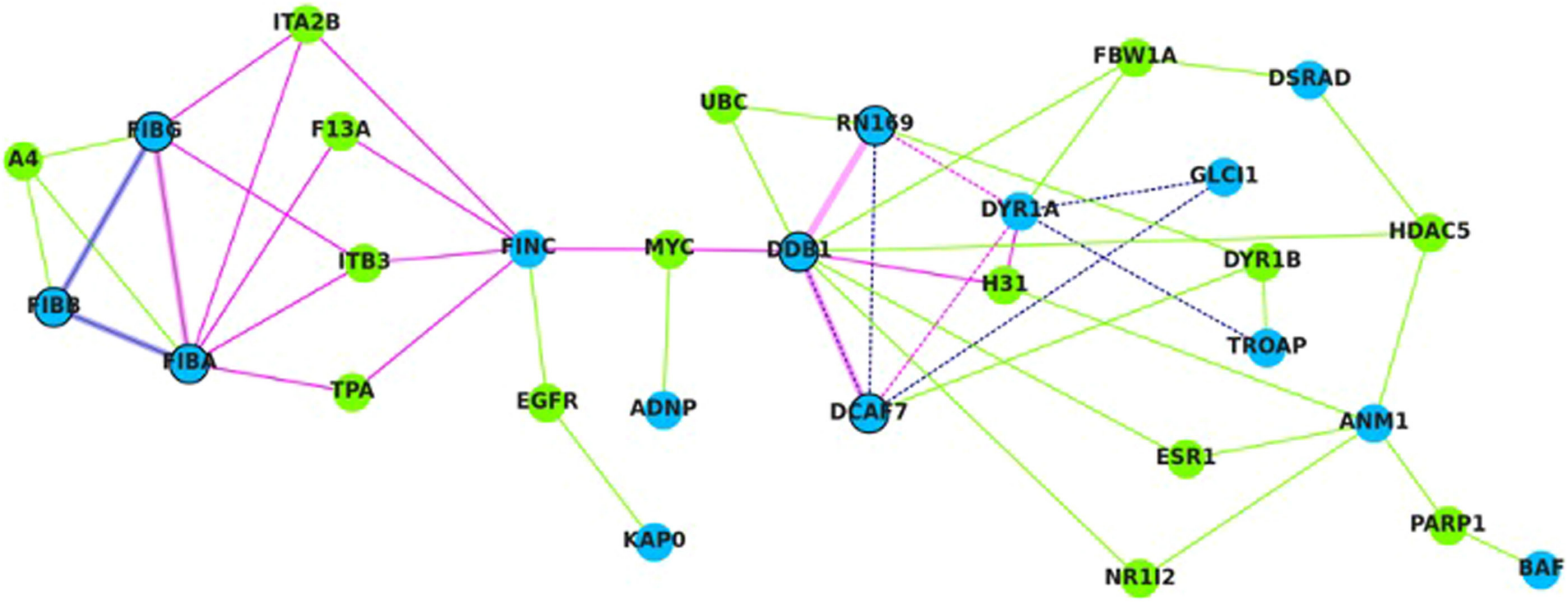
Graph representation of the Dyrk1A interactome. Blue and green nodes correspond to the input proteins (*n* = 21) and entries from BioGRID (*n* = 15), respectively. Thick edges and dashed edges correspond to interactions predicted by homologies and ELM analysis, respectively. The shortest path from Dyrk1A (DYR1A) to the fibrinogen (FIBA, FIBB and FIBG) is coloured in magenta.

To assess Dyrk1A interaction with proteins, coimmunoprecipitation (co‐IP) experiments were performed with proteins extracted from LCLs of three healthy individuals and three unrelated individuals with DS. The resulting Dyrk1A interactome includes FINC (the fibronectin), plasma fibronectin being an important self‐limiting regulator to prevent haemorrhage as well as excessive thrombus formation,^27^ but not the fibrinogen (Table S1). This may be due to a particularly indirect and/or weak Dyrk1A‐fibrinogen interaction that could not be captured in vitro. Therefore, to propose a molecular mechanism linking the Dyrk1A and fibrinogen levels, we performed a computational analysis that included (i) Dyrk1A, (ii) the 16 proteins that co‐precipitated with Dyrk1A and were detected by MS in at least two of the six LCLs, and (iii) the 3 *α, β* and *γ* fibrinogen chains. The list of 20 candidate partners was submitted to the Proteo3Dnet server for a preliminary analysis, which led to adding the undetected DNA damage‐binding protein 1 (DDB1) to the input list. A second analysis of the 21 proteins was then performed and a graph representation of the computed protein–protein interaction network was thus generated (Figure 1). Among the 21 input proteins, six remained as isolated nodes (data not shown). Among the 15 connected input proteins, only nine interacted directly with each other, into two distinct clusters. The first cluster is made of three fibrinogen chains (FIBA, FIBB and FIBG, respectively). The second cluster is composed of Dyrk1A (DYR1A), the glucocorticoid‐induced transcript 1 protein (GLCI1), the E3 ubiquitin‐protein ligase RNF169 (RN169), the trophinin‐assisting protein (or tastin, TROAP), the DNA damage‐binding protein 1 (DDB1) and the DDB1‐ and CUL4‐associated factor 7 (DCAF7). In this cluster, seven interactions are predicted as involving short linear motifs and are, therefore, potentially transient. The RN169‐DDB1 interaction, however, is predicted by homology (26.0% sequence identity) with two subunits found in the structure of the yeast spliceosome (PDB code 3JB9). The DDB1‐DCAF7 interaction is predicted both by the short linear motif analysis and homology: sequence identity <20% with subunits present in seven PDB structures (codes 6DNH, 6FBS, 6FUW, 6BLY, 6BM0, 6F9N and 6EOJ). Importantly, in the whole graph, all the interactions are confirmed by experimental data from BioGRID.

Despite the integration of data from BioGRID, PDB and ELM, no direct link could be found between Dyrk1A and the fibrinogen chains. Nevertheless, the additional nodes resulting from the BioGRID analysis, allowed establishing an indirect link. Thus, the shortest path between Dyrk1A and the fibrinogen passes through five other nodes: DCAF7, DDB1 and FINC, for the input proteins; MYC (the Myc proto‐oncogene protein) and ITA2B/ITB3/F13A/TPA for the nodes added to the network. No direct link was found in BioGRID between Dyrk1A and DDB1, nor between DDB1 and the fibronectin. It would be possible to connect Dyrk1A and the fibrinogen with only two degrees of separation, as direct interactions can be found in BioGRID between Dyrk1A and the fibronectin and between the fibronectin and the fibrinogen. However, as each of these connections are only supported by a single and high‐throughput experiment, the interactions were discarded from the network. In terms of subcellular location, the fibronectin‐fibrinogen interaction seems possible, as the former is secreted, and the latter is part of the extracellular matrix. However, this is not the case for the other interaction between the fibronectin and Dyrk1A, as the latter is found in the nucleus. Thus, the connection with intermediates such as DDB1 and DCAF7 seems realistic, as they are found in both the nucleus and cytoplasm. Finally, the fact that DDB1 and the fibronectin are linked through Myc is an issue. Indeed, this protein is described as exclusive to the nucleus and the computational analysis could not identify any intermediates. A possibility supported by experimental data from BioGRID could be that the Myc‐fibronectin interaction would be indirect, passing through the von Hippel–Lindau disease tumour suppressor (VHL), which is in the cytoplasm, membrane and nucleus, simultaneously. This hypothesis of an undetected interactant linking the Dyrk1A and fibrinogen levels would be consistent with the fact that there was no significant difference in protein levels, for the present co‐IP experiment, between healthy and DS LCLs (Wilcoxon rank‐sum test, with an *α* error of 5%). To conclude, with or without taking account of the subcellular location, Dyrk1A remains separated from the fibrinogen by at least six degrees.

### 3.2 Plasma fibronectin and fibrinogen levels are increased in mice over‐expressing Dyrk1A

We first quantified fibronectin and fibrinogen levels in plasma of mice overexpressing Dyrk1A. There are three independent mouse regions homologous to HSA21, the largest region is found on mouse chromosome 16 (MMU16) which contains the gene encoding Dyrk1A. We used the Dp (16)1Yey mouse model which carried the duplication of the entire MMU16 region syntenic to HSA21,^14^ and found an increased plasma fibronectin fibrinogen level compared to control (WT) littermate mice (Table 1). To show if the increased fibronectin and fibrinogen level is due to Dyrk1A overexpression, we used a transgenic line overexpressing Dyrk1A, the mBACtgDyrk1A mouse model.^13^ Fibronectin and Fibrinogen levels were increased in plasma of mice over‐expressing Dyrk1A (TgBACDyrk1A) compared to control (WT) mice (Table 1). Plasma fibronectin and fibrinogen are synthesized by hepatocytes and secreted into the blood plasma. We analysed the liver levels in mice over‐expressing Dyrk1A and found an increased fibrinogen content (9.4 ± 1.4 μg/mL versus 5.1 ± 1 μg/mL (*n* = 6 for each) (*p* < 0.04)) without difference in fibronectin content (data not shown). These results suggest that increased plasma fibrinogen levels would be due to increased hepatic production, the increased plasma fibronectin level being rather due to its increased half time in mice overexpressing Dyrk1A.

**TABLE 1:** Comparison of plasma fibrinogen and fibronectin levels obtained from male control mice (WT) and mice over‐expressing Dyrk1A (Dp (16) Yey and TgDyrk1A).

	WT littermate of Dp(16)Yey (*n* = 8)	Dp(16)Yey (*n* = 8)	WT littermate of TgDyrk1A (*n* = 8)	TgDyrk1A (*n* = 8)
Plasma fibronectin (ng/mL)	121.7 ± 16.2	232 ± 47.4*	121.2 ± 5	241.7 ± 42.2*
Plasma fibrinogen (μg/mL)	379.6 ± 48.5	609.7 ± 84.2*	304.2 ± 38	510.6 ± 66.8*

*Note*: Values are mean ± SEM of eight mice for each group. Statistical analysis was done with the Student's *t*‐test by using Statview software. *p < 0.05.

Even if the liver is the source of at least 98% of the circulating fibrinogen, expression, synthesis, and secretion have been in vitro described in a variety of non‐hepatic cells in response to inflammatory mediators.^28^ We therefore quantified plasma CRP and IL‐6 levels, CRP in plasma increasing during acute phase response to tissue injury or inflammatory stimuli especially IL‐6. No difference was found for plasma CRP (4.2 ± 0.2 ng/mL versus 4.4 ± 0.2 ng/mL (*n* = 6 for each)) and plasma IL‐6 (35.6 ± 1 pg/mL versus 40 ± 2.5 pg/mL (*n* = 6 for each)) levels in mice over‐expressing Dyrk1A compared to control mice.

### 3.3 Platelet fibrinogen levels are not modified in mice over‐expressing Dyrk1A

**FIGURE 2:**
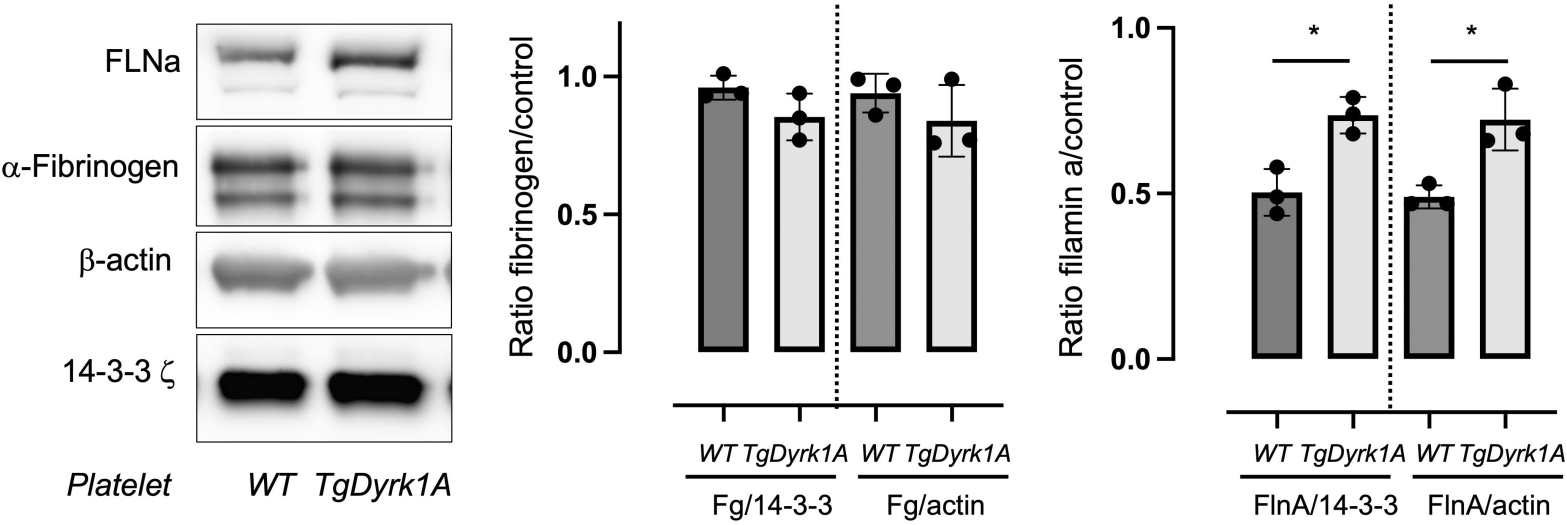
Comparison of platelet fibrinogen and control protein levels obtained from control mice (WT) and mice over‐expressing Dyrk1A (TgDyrk1A). Platelet lysates were prepared and Filamin A (FlnA), *α*‐Fibrinogen (Fg), *β*‐Actin, or 14–3‐3 z was determined by western blotting. Immunoreactive bands were visualized by chemiluminescence and quantified using a luminescent image analyser system. Data represent mean ± SD (*n* = 3) and results are expressed as fold increase relative to controls (*β*‐Actin, or 14–3‐3 z). Statistical analysis was performed using *t*‐test, *p < 0.05.

Platelet alpha‐granule fibrinogen is endocytosed from plasma. We therefore analysed the platelet levels in mice over‐expressing Dyrk1A and found no difference compared to control (WT) mice (Figure 2). There is therefore no increase in platelet fibrinogen endocytosis.

### 3.4 Platelet activation is normal in mice over‐expressing Dyrk1A

The next step was to investigate the impact of the overexpression of Dyrk1A on platelet function in vitro. In conditions of unstirred platelets after thrombin, ADP or Convulxin activation, no difference between TgDyrk1A platelets and WT platelets was observed for αIIbβ3 integrin activation and *α* granule secretion, measured by flow cytometry with a rat mAb JON/A(PE) specific for the activated conformation of the mouse integrin and a FITC‐labelled rat anti‐mouse CD62P mAb for P‐selectin expression (Figure S1 and S2) suggesting that the thrombocytopenia observed and the decrease bleeding time are not due to a thrombotic tendency in these mice. Moreover, no difference was observed in the expression level of the major receptors (αIIbβ3, GPVI, GPIbα and GPIbβ) analysed by flow cytometry (Figure S3).

Interestingly, cytoskeletal proteins were analysed. No difference was found for actin (Figure 2), but Filamin A level was increased in platelets of mice over‐expressing Dyrk1A. (Figure 2).

### 3.5 Platelet number is decreased in mice overexpressing Dyrk1A

**FIGURE 3:**
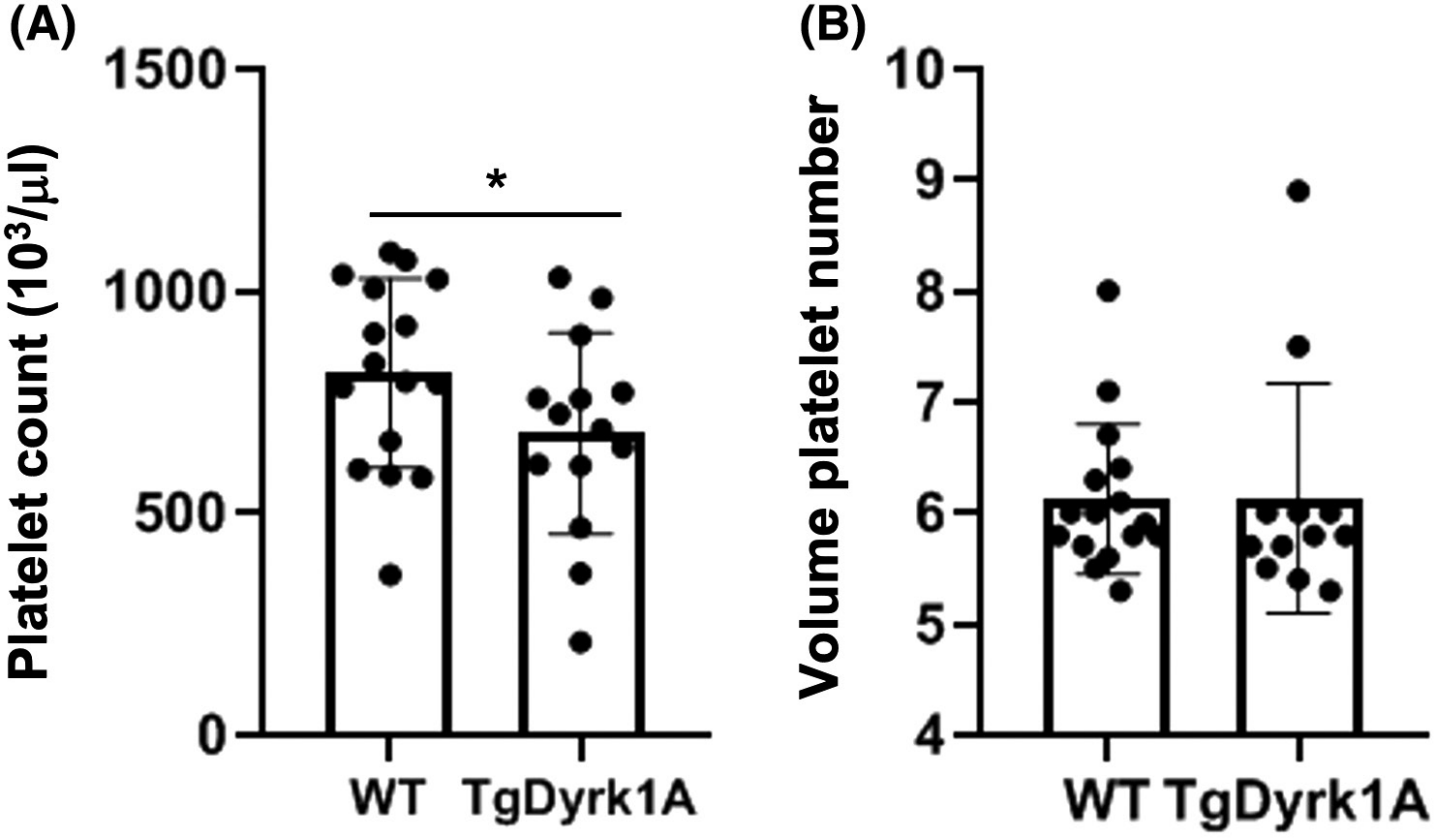
Platelet number is decreased in mice over‐expressing Dyrk1A. Platelets counts (A) and volume platelet number (B) were determined in control (WT) mice and mice over‐expressing Dyrk1A (TgDyrk1A). Data are presented as mean ± SEM and analysed with the Student's *t*‐test by using Statview software. *n* = 16 (eight males and eight females for each group). *p < 0.05.

To analyse the putative link between thrombocytopenia and over‐expression of Dyrk1A, blood was collected and analysed for platelet count (Figure 3). Mice overexpressing Dyrk1A (TgDyrk1A mice) have a significantly lower platelet number compared to control (WT) mice (Figure 3). However, no difference was found for the volume of platelets (Figure 3). No difference was also found for white blood cells, red cells, lymphocytes and haemoglobin levels. Furthermore, the platelet β‐galactose exposure was found like WT mice and no splenomegaly was detected (Figure S4). Taken as a whole, our data as well as the absence of abnormal levels of activated platelets detected in the circulation of TgDyrk1A mice (Figure S5) strongly support the conclusion that there is no accelerated clearance of Dyrk1A platelets.

### 3.6 Mice overexpressing Dyrk1A have a reduced bleeding time

**FIGURE 4:**
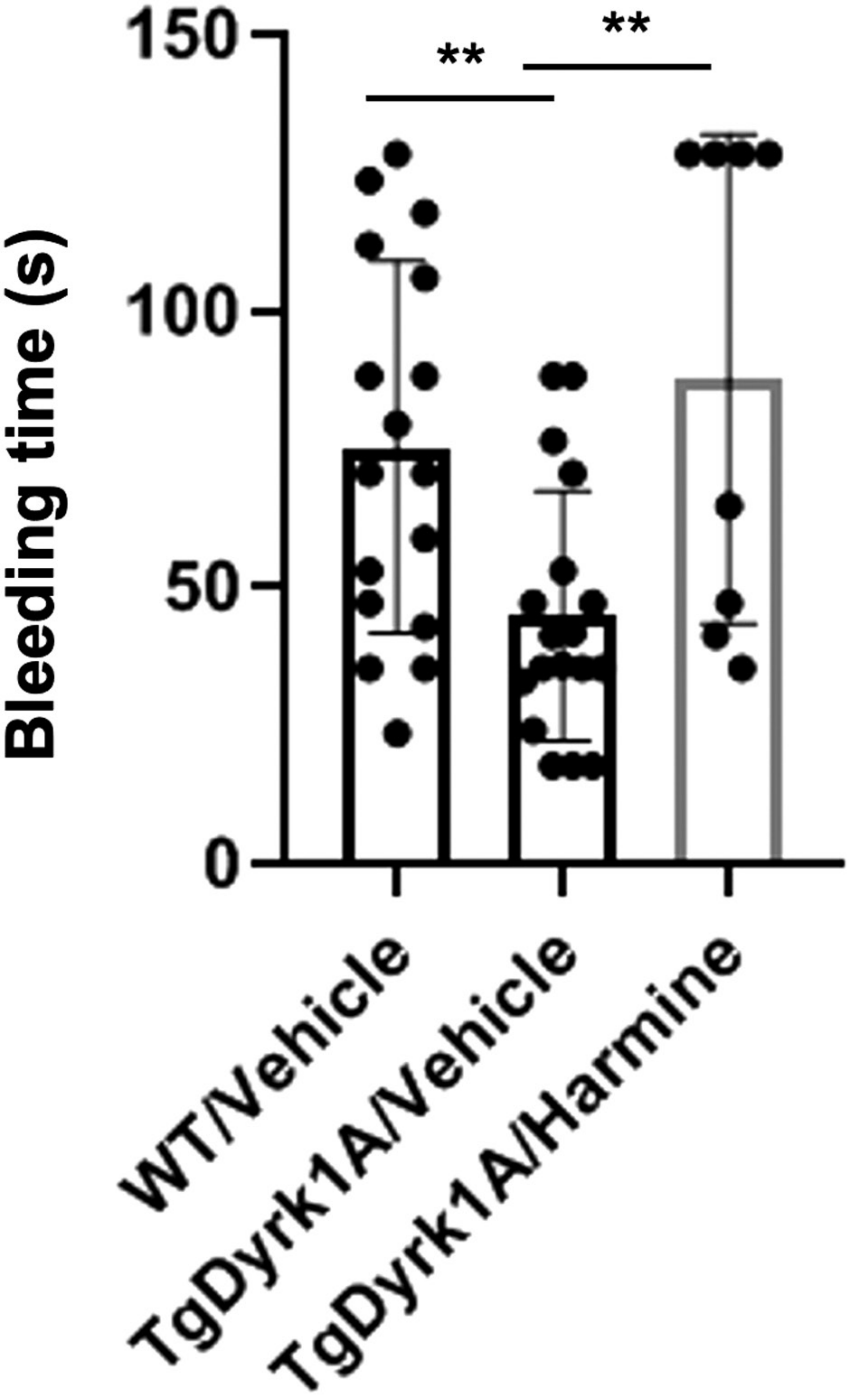
Tail bleeding time is decreased in mice over‐expressing Dyrk1A. Tail bleeding time was performed on male and female control (WT, *n* = 9 males and 9 females) or mice over‐expressing Dyrk1A (TgDyrk1A), with (TgDyrk1A/Harmine, *n* = 9 males and 9 females) or without (TgDyrk1A/Vehicle, *n* = 4 males and 4 females) harmine treatment. Data are presented as mean ± SEM and analysed with the Student's *t*‐test by using Statview software. **p < 0.005.

We next evaluated the consequence of Dyrk1A over‐expression on bleeding. For this, we determined the tail bleeding time of the TgDyrk1A mice (Figure 4). Mice over‐expressing Dyrk1A have a significantly reduced bleeding time compared to WT mice (Figure 4). To determine if the effects are dependent on Dyrk1A kinase activity, we treated mice with harmine, the most selective and potent inhibitor of Dyrk1A.^29^, ^30^ No effect was found in WT mice (data not shown), but harmine treatment prevents the decrease of bleeding time in TgDyrk1A mice (Figure 4). Taken together, these data indicate that increased Dyrk1A activity is associated with reduced bleeding time, which is in line with the increased level of fibrinogen observed, but not with the reduced platelet number.

### 3.7 Dyrk1A was detected in platelets

**FIGURE 5:**
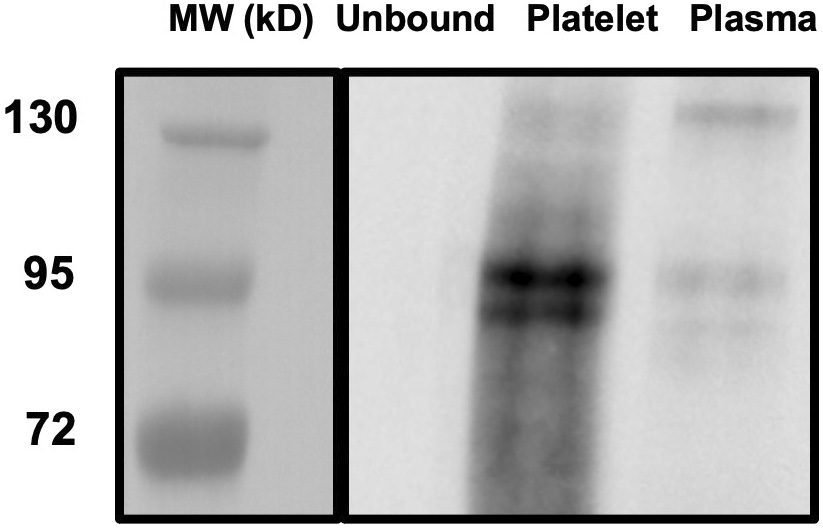
Dyrk1A is present in platelet. Western blotting showing Dyrk1A in human platelet and plasma after immunoprecipitation and immunoblotting with anti‐DYRK1A antibodies. Molecular Weight (MW) was visualized by colorimetry, immunoreactive bands were visualized by chemiluminescence.

As we found a discrepancy between platelet number and bleeding time in mice overexpressing Dyrk1A, we looked if we could detect the protein in platelets. We first analysed the platelet Dyrk1A protein levels in mice overexpressing Dyrk1A and found a 1.6‐fold increase compared to control (WT) mice (Figure S6). We previously detected Dyrk1A in human plasma by western blot analysis after immunoprecipitation.^23^ To confirm the presence of Dyrk1A in human platelets, we used the same method with human platelets to have large volume needed. Protein extracts from human platelets were subjected to immunoprecipitation with anti‐Dyrk1A antibody, and the presence of Dyrk1A in the immunoprecipitates was analysed by western blotting. Immunoblotting showed that Dyrk1A from platelets and plasma migrated at the same molecular weight (95 kD) (Figure 5). As previously demonstrated by genome‐wide platelet transcriptome and proteome analysis,^31^ Dyrk1A protein is not only present in plasma but also in platelets.

### 3.8 Plasma Dyrk1A results in part from platelet release

**FIGURE 6:**
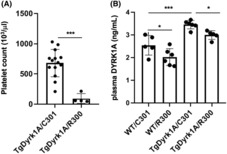
Plasma Dyrk1A results in part from platelet release. (A) Platelet depletion, after overnight injection of 1 mg/g of R300 antibody directed against GPIB or C301 antibody used as control, was verified by platelet counts. (B) Plasma Dyrk1A protein levels were determined in male and female control (WT) mice and mice over‐expressing Dyrk1A (TgDyrk1A). Data are mean ± SEM and analysed with two‐way anova followed by the Bonferroni/Dunnet post hoc test using Statview software. *n* = 6 (three males and three females for each group). *p < 0.05; ***p < 0.0001.

To analyse if plasma protein Dyrk1A results from platelet release, we quantified plasma protein Dyrk1A after platelet depletion in mice (Figure 6). For this, mice were injected with the R300 antibody directed against GPIB and C301 antibody as control. Platelet depletion was confirmed by platelet count (Figure 6A). Plasma Dyrk1A protein levels were decreased by 20% in control (WT) mice and mice over‐expressing (TgDyrk1A) (Figure 6B). These results show that plasma Dyrk1A, at least in part, result from platelet release.

## 4 DISCUSSION

In this report, we evaluated the effect of Dyrk1A overexpression on plasma fibronectin and fibrinogen levels, platelet number and half‐life, platelet receptors expression, platelet activation and bleeding in mice. Mice overexpressing Dyrk1A have a decrease in platelet count. It was previously shown that 66% of DS babies have mild to moderate thrombocytopenia.^3^, ^4^ Recent results showed that Dyrk1A kinase inhibition enhances platelet production in infantile megakaryocytes, identifying Dyrk1A as a critical morphogenetic brake in megakaryopoiesis.^32^ However, another study shows that the frequency of thrombocytopenia is 2.1% and thrombocytosis is much more frequent.^33^ Thus, the thrombocytopenia is far from being constant as expected from an overexpression of DyrK1A. Moreover, we only found a decrease platelet number by 25% in mice overexpressing Dyrk1A, this decrease being not due to splenomegaly, no accelerated platelet clearance with an unchanged platelet volume. Morowski et al induced different grades of thrombocytopenia in mice and found that tail bleeding time was unaffected by reductions of platelets up to 97.5%.^34^ Although thrombocytopenia is observed in some DS patients, the decreased platelet number is not sufficient to induce bleeding in DS patients.^3^, ^4^ In this sense, bleeding time, an in vivo assay designed to assess the ability of the mouse's haemostatic system to stop blood loss for a haemorrhagic problem, is reduced by 50% in mice overexpressing Dyrk1A, and harmine, a potent and selective inhibitor of Dyrk1A,^29^, ^30^, ^32^ prevents this decrease. The decreased bleeding time observed is not associated to an abnormal platelet receptors expression, to a defect of platelet activation, to the presence of activated platelets in the circulation or to an abnormal half‐life of the platelets. Moreover, the study of platelet activation by flow cytometry shows that mice overexpressing Dyrk1A do not have susceptibility to thrombosis. Nevertheless, we here demonstrate that Dyrk1A was not only detected in plasma, but also in platelet, plasma content resulting at least in part from platelet release. Dyrk1A is ubiquitously expressed in tissues with a stronger reactivity in central nervous system.^35^ Dyrk1A is associated with the dysregulation of neurotrophic pathways,^36^ particularly at the level of the brain‐derived neurotrophic factor (BDNF).^37^ In humans, BDNF has been known to accumulate in circulating platelets, and platelets can release it upon activation.^38^ In agreement with these results, we previously found that human plasma BDNF level correlates positively with Dyrk1A.^39^ Taken together, these results highlight the role of Dyrk1A in plasma and platelets.

At the molecular level, the computational analysis of the proteins that co‐precipitated with Dyrk1A demonstrate an indirect link between two clusters, one containing Dyrk1A and the other the fibronectin and the three fibrinogen chains (FIBA, FIBB, FIBG). Even if network analysis of Dyrk1A interactome revealed an indirect link between Dyrk1A, the fibronectin and the fibrinogen, fibronectin and fibrinogen levels was found to be increased in plasma of mice overexpressing Dyrk1A, linked to an increase of the fibrinogen hepatic production. However, there is no difference in platelet fibrinogen levels. Platelet *α*‐granule fibrinogen is derived from endocytic uptake, αIIbβ3 being the primary receptor mediated endocytosis.^40^ This result agrees with our results showing normal platelet receptors expression in mice overexpressing Dyrk1A. Sullivan and colleagues found that fibrinogen was downregulated in DS samples. Other genes localized on HSA21 must be therefore involved in the reduction of plasma fibrinogen level in DS patients.^12^

The first cluster including Dyrk1A is especially composed of the glucocorticoid‐induced transcript protein (GLCI1), tastin TROAP, the E3 ubiquitin‐proteine ligase RNF169 (RN169), DNA damage‐binding protein 1 (DDB1), WD repeat‐containing protein 68/WDR68 (DCAF7), with a direct interaction between Dyrk1A with RNF169 and WDR68. Previous results demonstrated that TROAP,^41^ RNF169^42^ and WDR68^43^ as cellular binding partners of Dyrk1A. The second cluster is composed not only of the fibronectin and the three fibrinogen chains (FIBA, FIBB, FIBG), but also of the integrin alpha‐IIb precursor (ITA2B), a protein receptor for fibronectin and fibrinogen, the integrin beta 3 (ITB3), a receptor for fibronectin which recognizes the fibrinogen gamma chain, these two proteins having a role in platelet adhesion and aggregation through binding of soluble fibrinogen,^44^ the factor XIII subunit A (F13 A), a blood coagulation factor^45^ and the tissue plasminogen activator (TPA).

Fibrinogen is also an active regulator of the inflammatory response. We previously demonstrated significant decreased concentrations of plasma alanine aminotransferase, a biomarker for diagnosis liver disease and reflecting liver damage, in plasma of mice overexpressing Dyrk1A, showing protective effect of increased Dyrk1A on liver function.^46^, ^47^ Here we found no difference in plasma CRP and IL‐6 levels. Therefore, increased plasma and hepatic fibrinogen levels do not seem due to a pro‐inflammatory effect of Dyrk1A in liver of mice. However, several pieces of evidence demonstrate a tight relationship between fibrinogen and inflammation in AD.^48^ The second cluster is also composed of the amyloid beta precursor protein/APP (A4). Cortes‐Canteli et al^49^ found that Aβ and fibrinogen interact, and their binding leads to increased fibrinogen aggregation, Aβ fibrillization and the formation of degradation‐resistant fibrin clots. Dyrk1A interacts with APP and APP processing, thus promoting the pathological Aβ pathway and the production of Aβ,^50^, ^51^ thus playing an important role in AD pathogenesis. Taken together, our results underline the role of Dyrk1A and fibrinogen on dysregulation of haemostasis in AD physiopathology. Even if no difference was found in platelet fibrinogen content, mice overexpressing Dyrk1A have increased platelet Filamin A level. Data from platelets underline how Filamin A integrates signalling pathways between the plasma membrane and the actin cytoskeleton, platelet function being directly contingent on actin cytoskeleton integrity.^52^ Previous data showed an enhancement of Filamin A level in control neonatal megakaryocytes subjected to Dyrk inhibition.^31^ SRF/MKL1A is the key co‐activator of many actin cytoskeletal genes,^53^ and unlike the results obtained for the Filamin A level, we found no difference in βactin level. Therefore, Filamin A regulation in platelets from mice over‐expressing Dyrk1A would not be link to SRF/MKL1A pathway. Filamin A is also a key partner in Aβ and Tau pathological processes in AD.^54^, ^55^

## 5 CONCLUSIONS AND LIMITATION

Our results indicate that overexpression of Dyrk1A in mice induces decreased bleeding consistent with increased plasma fibronectin and fibrinogen levels, revealing a new role of Dyrk1A depending on its indirect interaction with these two proteins. Dyrk1A would be one of the proteins involved in hematologic disorders of DS patients, but other proteins linked with DS aneuploidy could be involved. Hemostatic parameters will have to be therefore studied in DS people.

## AUTHOR CONTRIBUTIONS

**Cecile Loubière:** Methodology (equal); writing – review and editing (equal). **Guillaume Postic:** Formal analysis (equal); methodology (equal); writing – review and editing (equal). **Janany Kandiah:** Formal analysis (equal); methodology (equal); writing – review and editing (equal). **Jaysen Sawmynaden:** Formal analysis (equal); methodology (equal); writing – review and editing (equal). **Marie Vilaire:** Resources (equal); writing – review and editing (equal). **Thibaut Léger:** Formal analysis (equal); methodology (equal); writing – review and editing (equal). **Jean‐Michel Camadro:** Formal analysis (equal); resources (equal); writing – review and editing (equal). **Eric Bun:** Methodology (equal); writing – review and editing (equal). **Gautier Moroy:** Conceptualization (equal); data curation (equal); resources (equal); validation (equal); writing – review and editing (equal). **Olivier Christophe:** Conceptualization (equal); data curation (equal); validation (equal); writing – review and editing (equal). **Nathalie Janel:** Conceptualization (equal); data curation (equal); funding acquisition (lead); project administration (lead); writing – original draft (lead); writing – review and editing (equal). **Jean Solarz:** Methodology (equal); writing – review and editing (equal). **Frederic Adam:** Methodology (equal); writing – review and editing (equal). **Daniella Balduino Victorino:** Resources (equal); writing – review and editing (equal). **Marie Claude Potier:** Resources (equal); writing – review and editing (equal). **Alexandre Kauskot:** Conceptualization (equal); data curation (equal); validation (equal); writing – review and editing (equal).

## CONFLICT OF INTEREST STATEMENT

No potential conflict of interest was reported by the authors.

## Supporting information


Figure S1:

Figure S2:

Figure S3:

Figure S4:

Figure S5:

Figure S6:

Table S1:

## Data Availability

Data available on request from the authors.
