# Supplementary figures and images for: Over‐expression of Dyrk1A affects bleeding by modulating plasma fibronectin and fibrinogen level in mice
Source: J Cell Mol Med. 2023 Jul 6;27(15):2228–38. doi: 10.1111/jcmm.17817 (PMC10399536; doi:10.1111/jcmm.17817)

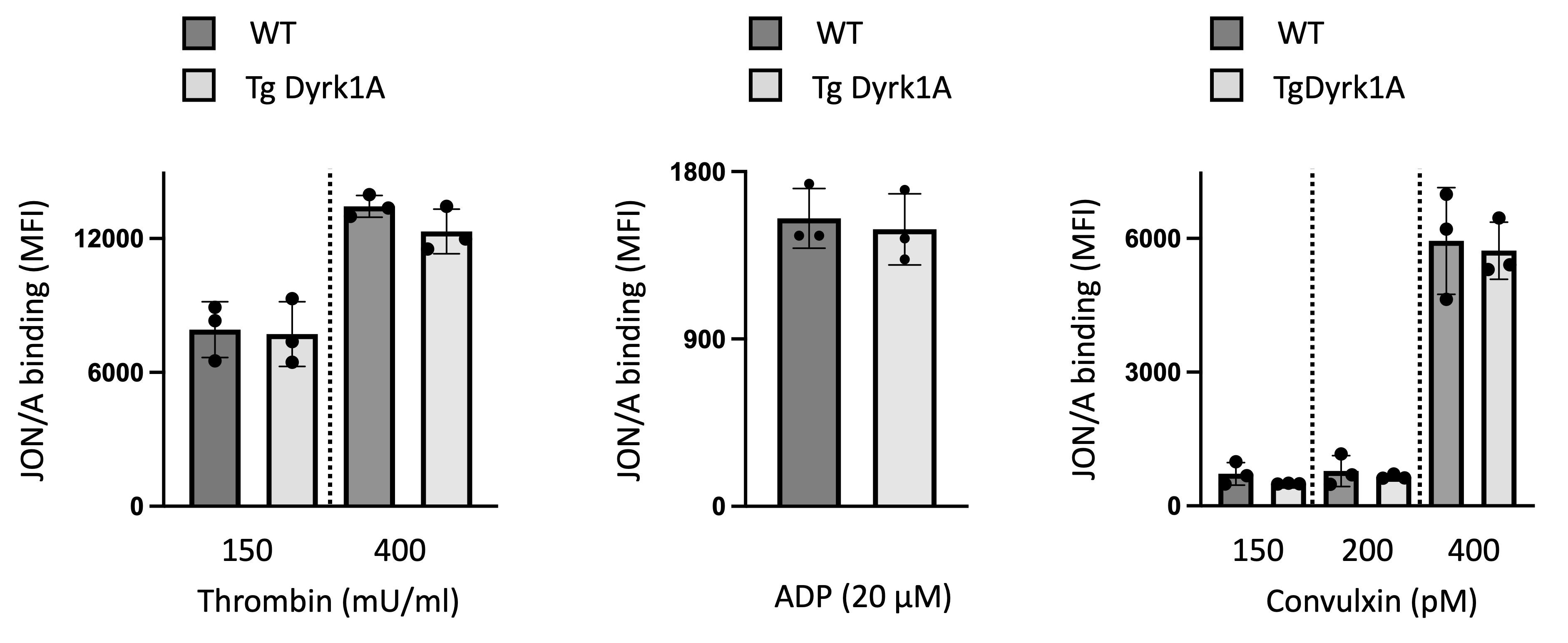

Supplement: Supplementary file 1 — Figure S1: [file JCMM-27-2228-s004.jpg]

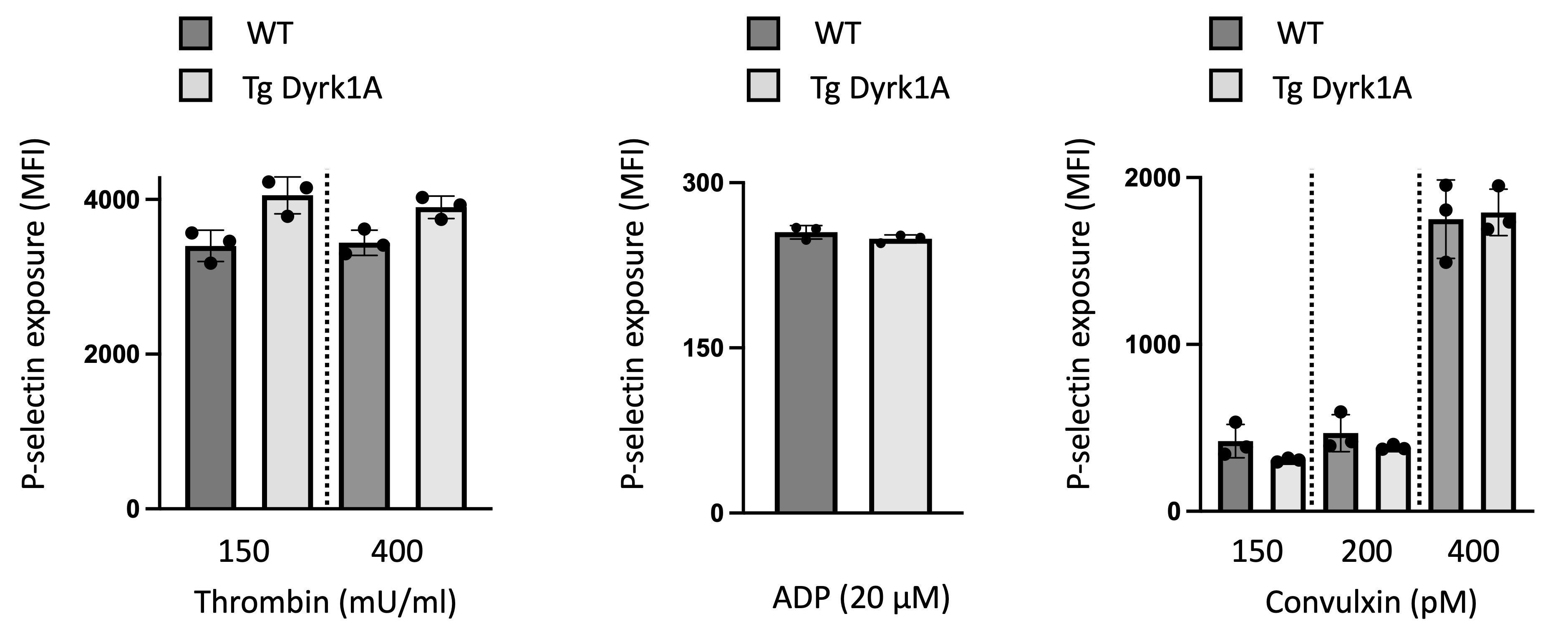

Supplement: Supplementary file 2 — Figure S2: [file JCMM-27-2228-s003.jpg]

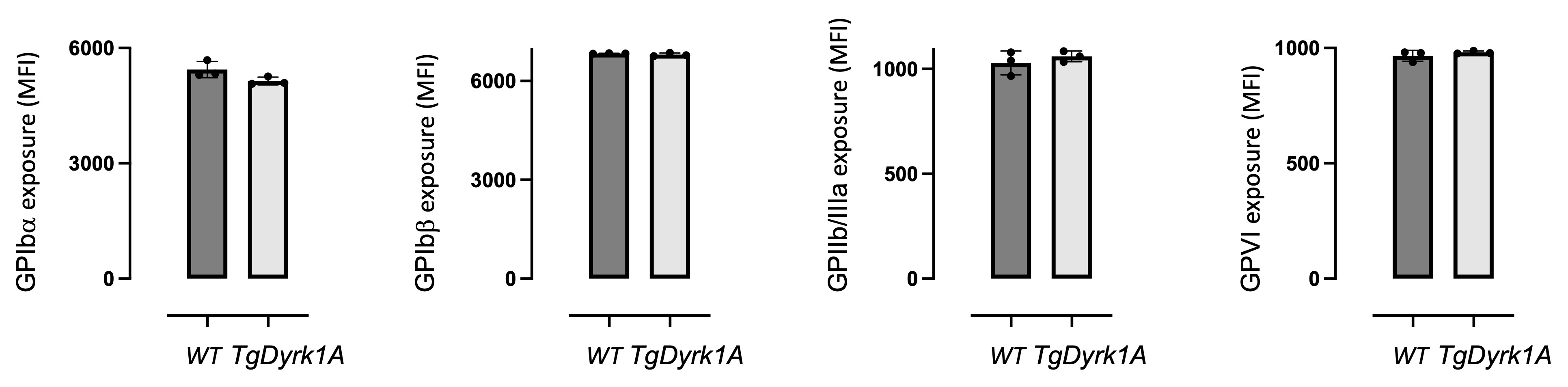

Supplement: Supplementary file 3 — Figure S3: [file JCMM-27-2228-s005.jpg]

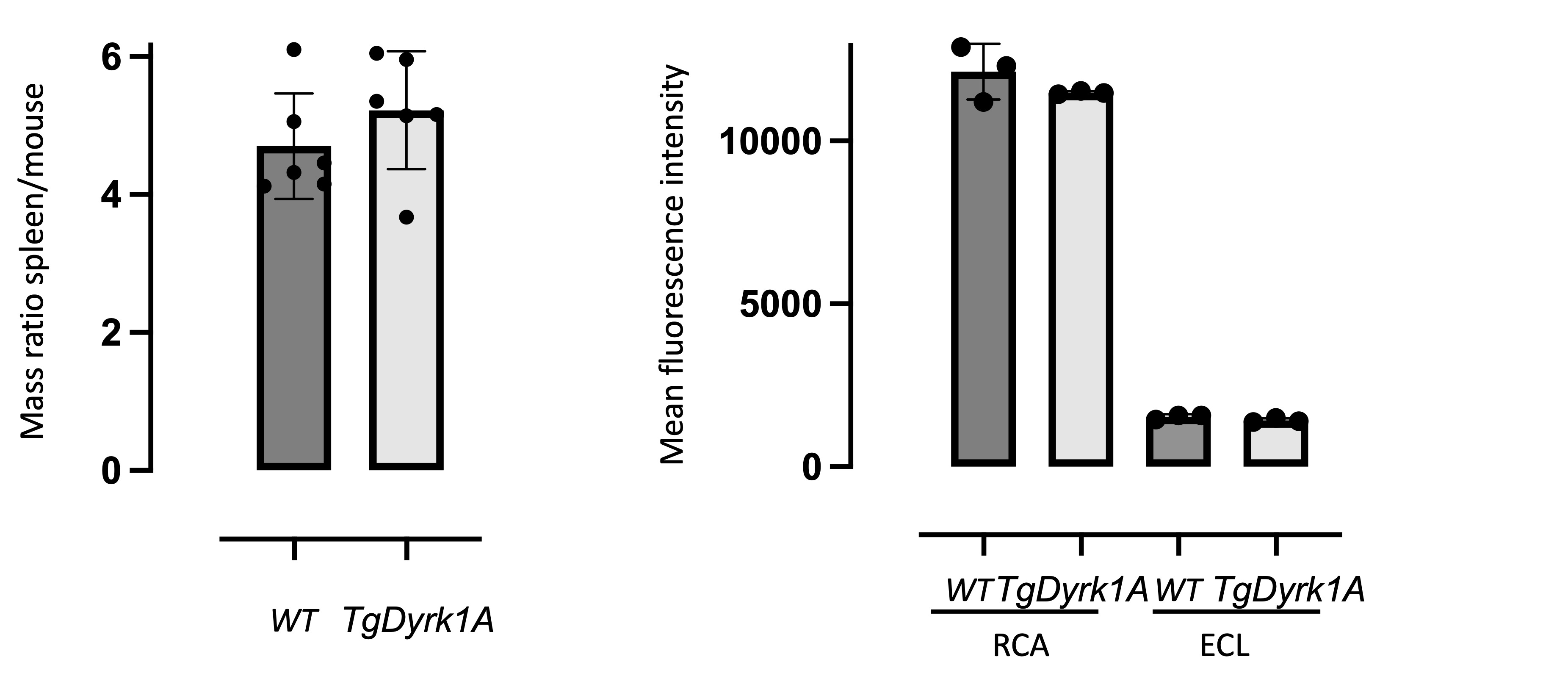

Supplement: Supplementary file 4 — Figure S4: [file JCMM-27-2228-s006.jpg]

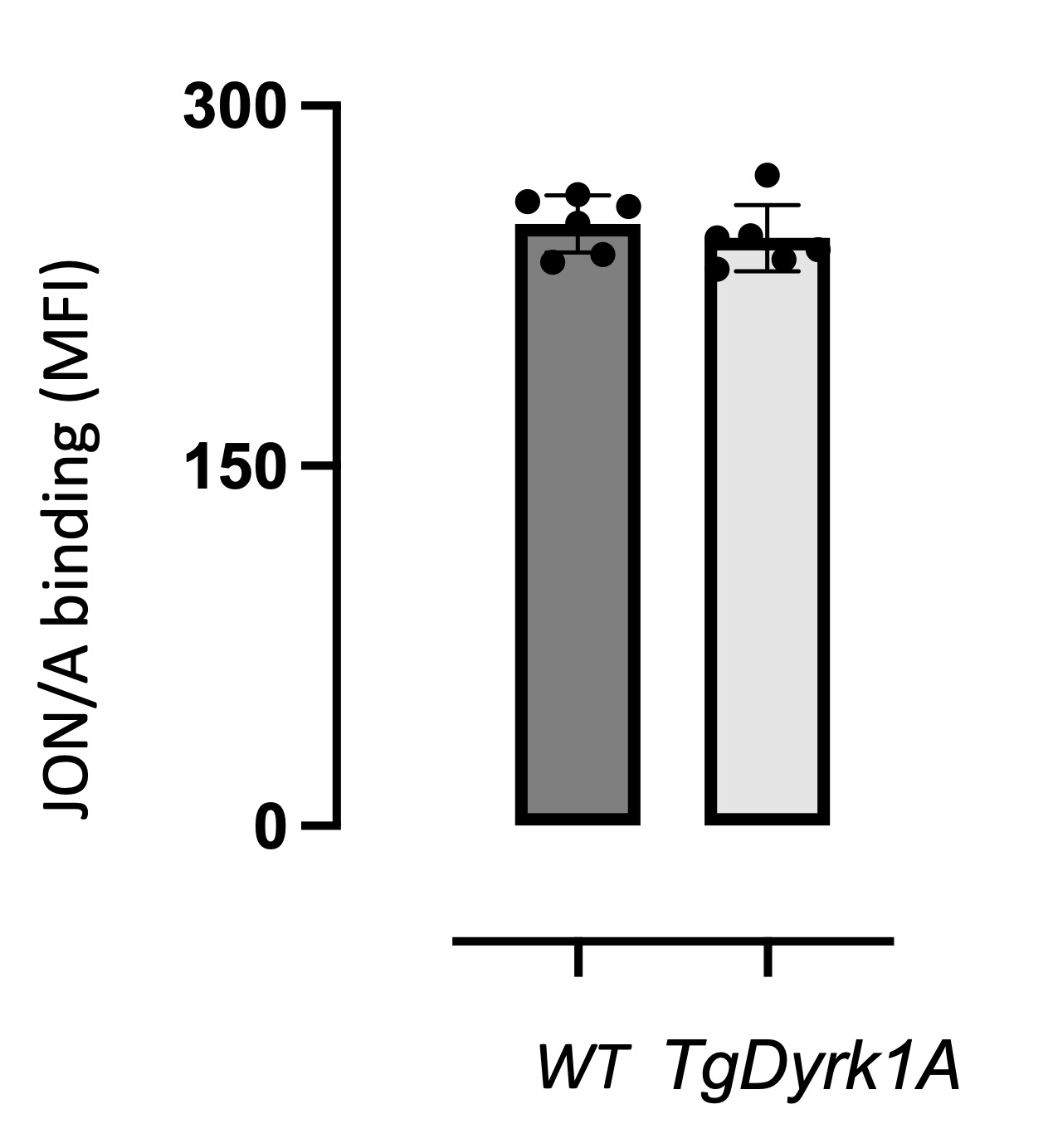

Supplement: Supplementary file 5 — Figure S5: [file JCMM-27-2228-s007.jpg]

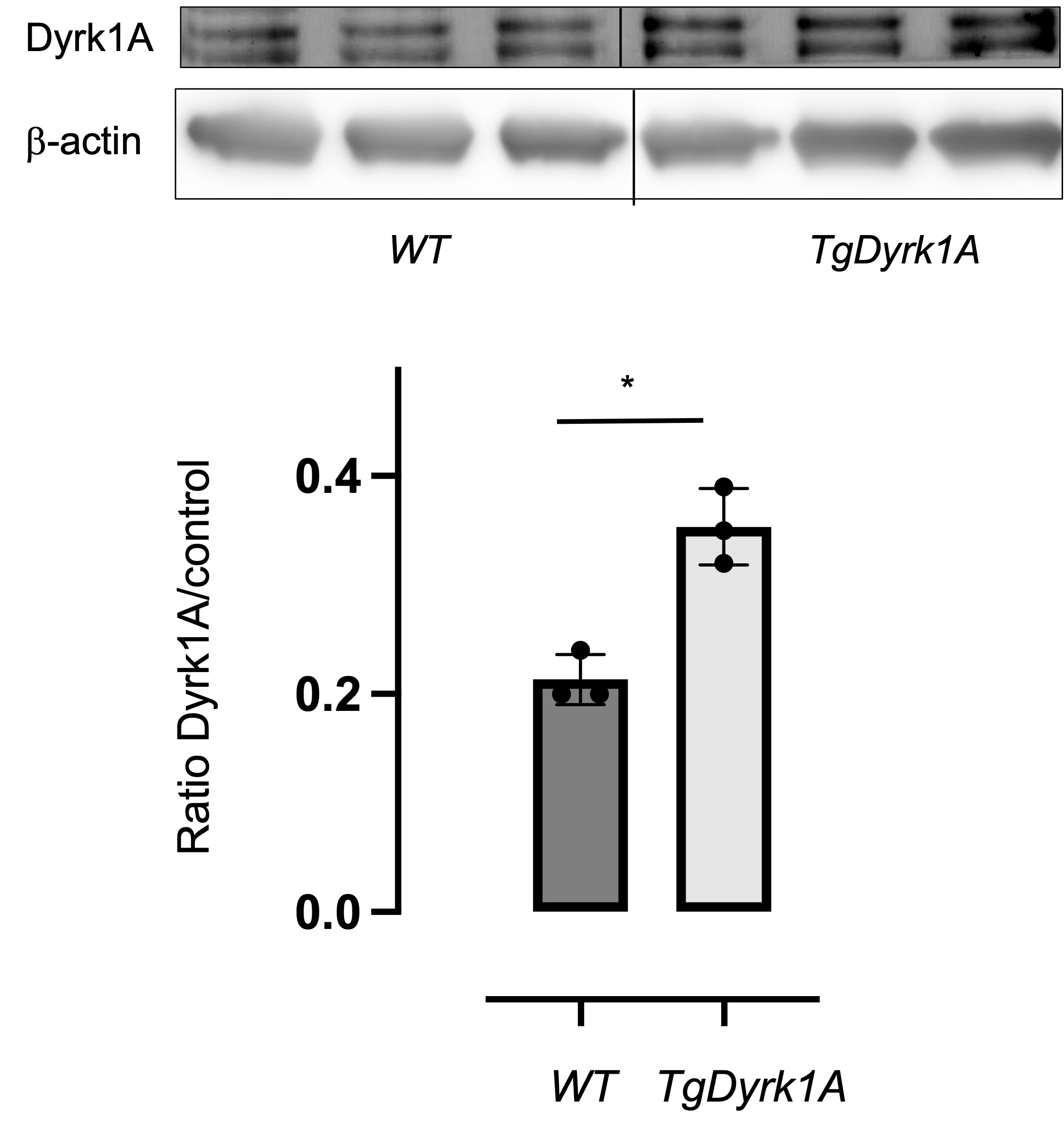

Supplement: Supplementary file 6 — Figure S6: [file JCMM-27-2228-s001.jpg]

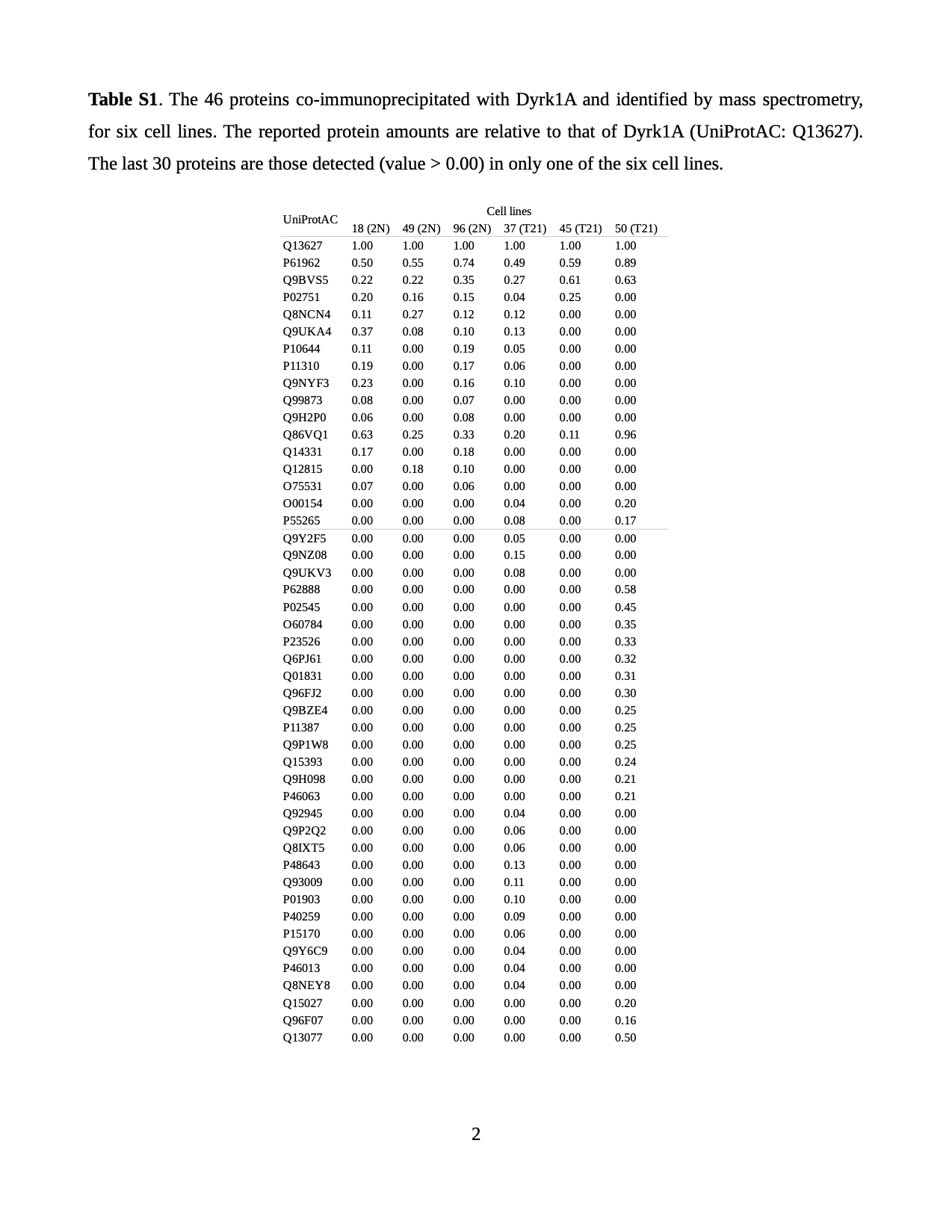

Supplement: Supplementary file 7 — Table S1: [file JCMM-27-2228-s002.tiff]
